# Genetics and genomic medicine in Israel

**DOI:** 10.1002/mgg3.73

**Published:** 2014-03-13

**Authors:** Joël Zlotogora

**Affiliations:** Department of Community Genetics, Public Health Services, Ministry of Health and the Hebrew UniversityJerusalem, Israel


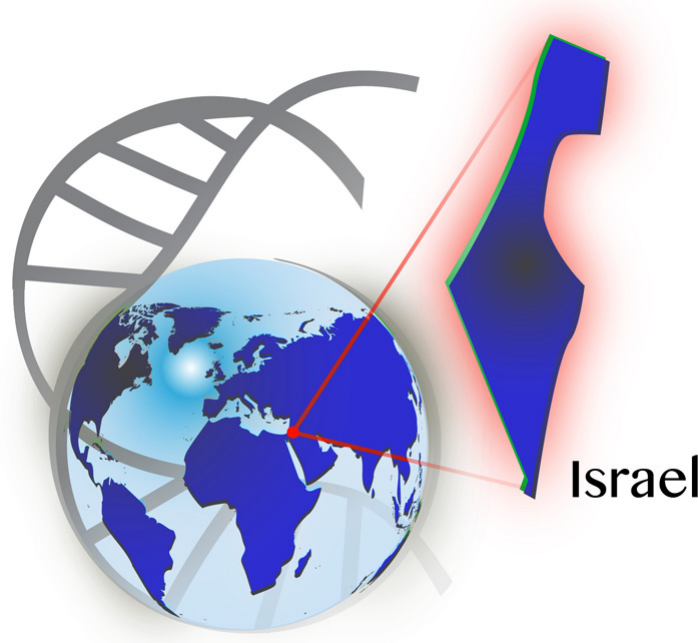


The State of Israel that was declared independent in 1948 is a country that is ∼450 km in length and 180 km at its maximum width (Fig. 1). At the end of 2012 there were 7,984,500 inhabitants in Israel among which 5,999,600 (75.1%) were Jews, 1,387,600 (17.4%) Muslim Arabs, 158,400 (2.0%) Christian Arabs, and 131,500 (1.6%) Druzes (Statistical Abstracts of Israel 2013). In 2012, there were 170,940 live births among which 125,409 (73.4%) were Jews, 36,041 (21.1%) Muslim Arabs, 2610 (1.5%) Christian Arabs, and 2371 (1.4%) Druze.

**Figure 1:**
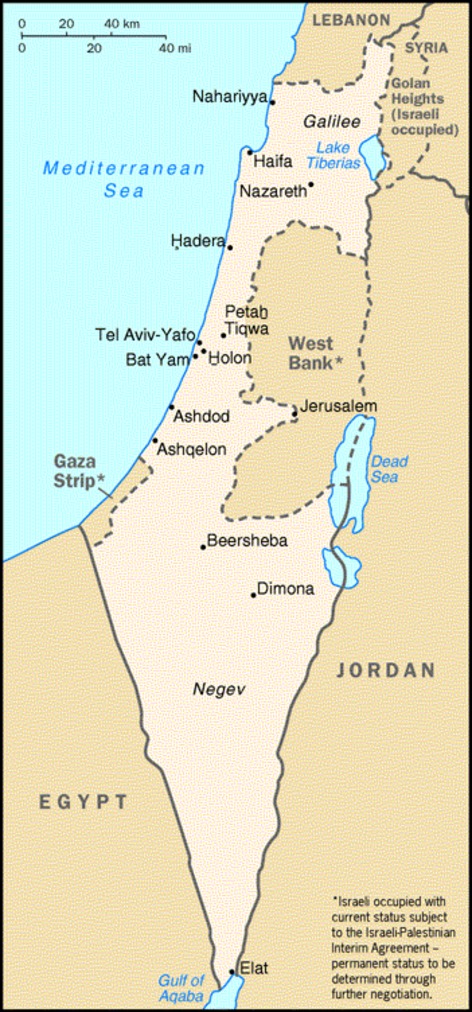
Map of Israel.

Most of the population lives in the coastal area and, in particular, in the Tel Aviv region. The Jewish population is located primarily in cities, whereas the Arab population mostly resides in small towns and villages.

## The Jewish population

The Jewish people originate from the Middle East. After the destruction of the First Temple, part of the community remained in Palestine while another part moved eastward founding the Babylonian Jewry, ancestors of the Iraqi and Iranian Jews. With the rise of the Greco-Roman empires, Jews began to move also westward as far as Spain and Portugal, where a large Jewish community developed in the Middle Ages. Sephardic Jews are descendants of the Jews who were forcibly expelled during the Inquisition in Spain and settled mostly in the countries along the Mediterranean Sea and in the Netherlands and the New World. The major movement of Jews toward Eastern Europe was in the middle Ages to France and Germany. Later on, there were two important waves of migrations of the Ashkenazi Jews in central Europe: the first in 15th to 16th century eastward to Bohemia and eventually to Poland-Lithuania and then from the end of the 18th century back to the west (Germany, Netherlands, England, and USA).

The Jewish communities differed in their cultural outlook and way of life, in their spoken language and their traditions. While differences existed between the various regions where the Ashkenazi Jews were living, it is difficult to delineate subgroups among them. On the other hand, other Jewish communities remained geographically separated one from the other and developed as distinct identities. Therefore, the non-Ashkenazi Jews are best delineated as communities according to their country/region of origin. While the non-Ashkenazi communities represented more than 90% of the Jews in the 12th century, because of the size expansion of Ashkenazim, the non-Ashkenazi Jews represented only 10% of the world Jewry in 1930. The total number of Jews, in particular Ashkenazi Jews, was dramatically reduced by the holocaust but today, the estimated number of Jews worldwide is 13–14 millions. In 2012, there were 5,999,600 Jews living in Israel, ∼50% were of Ashkenazi origin (Statistical Abstracts of Israel 2013).

Consanguineous marriages are allowed in the Jewish religion and they were common in all the communities including the Ashkenazi Jews. In a survey in Israel of Jewish women interviewed after delivery in maternity wards throughout Israel in 1956 soon after the foundation of the State and therefore representing the situation in the countries of origin (Goldschmidt et al. 1960), the consanguinity rate varied from 2.5% among Ashkenazi Jews to 28.7% among Jews from Iraq (Table 1). In a follow-up study performed in 1991 it was demonstrated that intercommunity marriages became common and within each of the communities consanguineous marriages became less frequent (Cohen et al. 2004).

**Table 1:** Type of marriages among Jews in two different periods in Israel (Cohen et al. 2004)

	1956		1991	
	Related (%)	Intracommunity (%)	Related (%)	Intracommunity (%)
Ashkenazi	2.5	90.2	1.2	72.1
Iraq	28.7	59.4	3.2	31.9
Libya	10.7	69.1	2.3	25.8
Morocco	10.7	69.1	1.9	48.3
Iran	26.2	50.1	9.2	43.2
Syria	8.1	49.3	5.1	5.1
USSR^1^	16.9	33.7	5.8	83.5
Yemen	18.3	61.8	6.2	61.7

1 Non-Ashkenazi, the survey in 1991 was done few years after a large wave of immigration from the late USSR.

The first observations that various genetic diseases are relatively frequent among Jews were made among Ashkenazi Jews in Europe and in the United States probably because of the size of the community and the relative advance in medicine in the countries in which they lived. Soon after the foundation of the state, several Israeli physicians in particular the late Haim Sheba and the late Rileftd Goodman initiated studies on genetic diseases among the various Jewish communities (Goodman 1979). Since then the molecular basis of most of the disorders relatively frequent among Jews have been elucidated and many of the mutations were identified.

Some of the disorders like thalassemia, Familial Mediterranean Fever (FMF), or G6PD deficiency found with a relatively high frequency among some of the Jewish communities are also frequent in the non-Jewish local population. However, these are exceptions and in most cases the high frequency of the genetic diseases is unique to the Jewish community as a result of its religious isolation. In most of the cases a relatively high frequency is found in a single Jewish community due to a unique founder mutation. There are few diseases in which the same mutations are found in several communities either since they were ancient mutations and existed already before the dispersion of the Jews or because of migration form one community to another (Zlotogora et al. 2000).

## The non-Jewish population

The non-Jewish Israeli citizens include Muslim Arabs, Christian Arabs and Druzes representing 17.4%, 2%, and 1.8% of the Israeli population, respectively. The Bedouin-Arabs, residing mostly in the Negev desert, comprise ∼250,000 individuals, representing 15% of the Muslim Arabs in Israel. There are also some smaller ethnic groups in Israel in particular the Circassians, Armenian, and Samaritains.

The Arabs and Druze are generally living in villages/tribes which were founded only by a few individuals less than 10 generations ago. There are more than 100 entirely non-Jewish localities in Israel, with 88 having more than 2000 inhabitants. Most of the localities include less than 20,000 inhabitants a few are larger but still almost all with less than 50,000 individuals (Statistical Abstracts of Israel 2013). In most cases the locality includes only one ethnic community but sometimes the population is mixed. A small proportion of the Arab population lives in towns with a Jewish majority. Most of the Bedouins from the north and left are living in similar conditions as the whole Arab population. In the Negev, more than 60% of the Bedouins live in Bedouins townships, most of the remaining resisted sedentarization and urban life.

Similarly to the Arab population in the Middle East, consanguineous marriages are frequent among the Israeli Arabs as well as the Druze with a preference for first cousin marriages. In more than 25% of Muslim Arabs and Druze marriages the spouses are first cousins with an additional 20% related in other ways (Vardi-Saliternik et al. 2002). Among the Muslims, consanguineous marriages are the most frequent among the Negev Bedouins (35% of marriages first cousins and 34% related). Consanguinity is less frequent among Christian Arabs (21% of marriages first cousins and 11% related).

Many autosomal recessive diseases have been diagnosed among the Israeli Arabs and Druze, with several being relatively frequent in the whole population such as thalassemia, FMF, or deafness (Zlotogora 2010). Other autosomal recessive diseases are rare in the general population but are frequent in one of the communities either in a large kindred, a village, a region, or even in some cases in a whole community. Most of the rare diseases are present in one community only. In most of the diseases found in more than one community, different mutations have been leftacterized but there are some exceptions, some being ancient mutations (Zlotogora 2010).

## The Israeli National genetic database

In order to improve the availability of the existing knowledge about the genetic disorders in the Israeli population and their distribution in the various Israeli groups, the Israeli national genetic database (available at http://server.goldenhelix.org//israeli/), was launched in 2006 (Zlotogora et al. 2007ab2007b). One of the features includes short clinical summaries that are divided into two parts, one for disorders present among Jews and the other for disorders among non-Jews. Another feature of the database includes data on mutations for autosomal recessive diseases and founder dominant or X-linked mutations. It is possible to search the database for mutations by disorder or by population, classified as either Jews or non-Jews. The Jewish population is subdivided as Ashkenazi or according to the country of origin, while the non-Jewish population is subdivided according to religion. Muslim Arabs have been separated in two groups, Muslims (non-Bedouins) and Bedouins. Results are returned in a tabular format, in which the gene and mutation(s) are given in their official nomenclature, accompanied by the allelic and carrier frequencies, where available, and the respective OMIM number, hyperlinked to the corresponding web page. On 1 January 2014 there was a total of 1528 entries, 724 among Jews and 804 in non-Jews. Another feature allows obtaining knowledge about the genetic diseases existing in each of the localities where Arabs and Druze are living. In this part of the database, the monogenic disorders known in each locality are included even if no molecular data are available (1043 entries on 1 January 2014). In order to protect privacy and to ensure anonymity, data access is provided only to geneticists on the basis of a username and password. The search may be done either by locality or by disorder. This feature allows obtaining a list of the disorders known in the locality or the distribution of a disorder in the different communities and among the different localities. Another feature of the database is about the molecular genetic laboratories accredited by the Ministry of Health. The information includes the type of tests that are provided by the laboratory including details about the mutations examined and/or other services such as linkage or sequencing. Results are returned either by laboratory or by test, allowing knowing where a specific genetic test is done in Israel.

## Ethical, Legal, and Social Implications of Genetic Testing in Israel

### Genetic information law – 2000

The purpose of the law is described in its introduction: “to regulate the conducting of genetic testing and the provision of genetic counseling, and to protect the right to privacy of the person subject to such testing in respect of identified genetic information, but without derogating from the quality of the medical treatment, medical and genetic research, the advancement of medicine and the protection of public welfare.” The law defines a “genetic test” as a test performed to determine sequence of human DNA. The law deals with all the aspects of the genetic tests from the genetic testing laboratories, the information that must be given before and after any genetic test and the different individuals who may give genetic counseling in Israel. One of the bases of the law is that genetic tests must be done in laboratories accredited by the Ministry of Health or, in other words, Israeli laboratories; however, the Ministry of Health allows the possibility to perform tests outside the country when necessary after special authorization. Another aspect of the law is the interdiction to conduct in minors either genetic tests for reproductive purposes or presymptomatic tests without immediate implications. In addition to physician medical geneticists and genetic counselors, the law authorizes MDs to give genetic counseling in the field of their specialty. The law also forbids discrimination at work or for insurance on the basis of genetic tests.

While Israel was among the first countries in which a genetic law was promulgated, the law needs to be updated and discussions about its revision begun. The major change concerns the definition of genetic tests which is planned to be changed to include all the tests providing genetic information on the individual.

### Prenatal diagnosis and therapeutic termination of pregnancy

The approaches to genetic testing are diverse in the Israeli Jewish population, mainly because of the differences in the degree of religiosity and traditions from the countries of origin. The ultraorthodox community that represents slightly more than 10% of the Israeli Jewish population is a relatively homogeneous group as for the way it uses genetic tests. Strict Judaism permits abortion only in cases where continuing the pregnancy would put the mother's life in serious danger, but each case is considered and decided independently by the family's Rabbi. Therefore, as a rule the ultraorthodox Jews the preference is for premarital genetic testing (Dor Yeshorim) and not participating in pregnancy screening or testing. In this community, preimplantation genetic diagnosis (PGD) is the preferred procedure for couples at risk for genetic diseases, since the embryo is considered an individual only 40 days after conception. The majority of the Israeli Jewish population, even if religious (but not ultra orthodox as mentioned above), will envisage termination of pregnancy at almost every stage of the pregnancy if the fetus is abnormal. The Israeli law allows for termination of pregnancy at all stages of pregnancy on the condition that the fetus is affected with a severe disease as determined by a medical committee. As a result of this duality, in the last decade most Jewish children with Down syndrome are born to women from the ultraorthodox community (Zlotogora et al. 2007a).

According to the Islamic Law, interruption of pregnancy is allowed if it puts the mother's life in serious danger. However, according to several religious authorities in case of a severe disease of the fetus, interruption of pregnancy may be allowed before the 120th day of the pregnancy and religious rulings (fatwa) have been issued in various countries (Hessini 2007). The Arab Muslim population of Israel is mostly religious/traditional and interruptions of pregnancy are relatively rare while the utilization of noninvasive tests that are recommended is high (Zlotogora et al. 2007a).

## Health Services

### General

Since the enactment of universal health care law in 1995, the entire population benefits from a comprehensive health insurance plan that included curative and preventive out-patient care as well as hospital care (Chemke and Zlotogora 1997). In addition more than 80% of the population has a complementary insurance either provided by the heath funds or private. The Ministry of Health, besides the supervision and planning of the provision of health services, is also a direct health care provider through governmental hospitals and other curative and community maternal and child health clinics. The Clalit health fund is responsible for the provision and delivery of health care to some 50% of the population. The three other health insurance funds, Maccabi, Leumit, and Meuhedet, are responsible for the remaining of the population. In addition, there are voluntary nonprofit institutions like the Hadassah Medical Organization and a few religious and some missionary hospitals.

### Genetic services in Israel

#### Genetics in hospital setting

Even though medical genetics had been practiced by a number of physicians and scientists for several years, the first genetic counseling clinic opened in Jerusalem by the late Tirza Cohen in 1964 (Chemke and Zlotogora 1997). Since then, medical genetics departments/units have been established in almost all the hospitals and all are affiliated with one of the five medical schools. Medical genetic services in Israel are organized in comprehensive independent departments/units of different sizes within the hospitals. In 15 of the public hospitals and one private hospital there is a medical genetic department including a genetic counseling clinic and a cytogenetic laboratory that offer prenatal diagnosis. A molecular genetic laboratory is an integral part of 14 of these medical genetic departments. There are, in addition, two genetic units that were recently created in public hospitals that include a genetic counseling clinic and a molecular genetic laboratory and that are sending cytogenetic tests including amniotic samples to other genetic departments.

In all hospital medical genetic department/units, there is always at least one Israeli board certified physician medical geneticist and at least one genetic counselor. The repartition of the different area of the counseling is different in each of the departments. Prenatal clinics are found in all the departments and cancer genetics clinics also exist in most. In addition, special joint clinics have been opened for particular genetic diseases such as ophthalmology, craniofacial disorders, and perinatal clinics in which geneticists are part of a team that includes other specialists. The genetic team is also involved in counseling and diagnosis of hospitalized patients.

There are six PGD units in Israel (four in the region of Tel Aviv and two in Jerusalem); each unit is a component of the IVF division and of the genetic department and offers molecular and cytogenetic diagnosis. The indications for PGD are delineated by the Ministry of Health. Using PGD for sex selection for nonmedical purposes is forbidden in Israel. A special committee exists and is entitled to make exceptions in rare cases.

#### Genetic counseling in the community

Most health funds have genetic clinics run by medical geneticists that provide genetic consultations which numbers are rapidly increasing. In addition, several genetic clinics in the community are affiliated with the regional hospital genetic department, and are run by a member of the genetic department team.

### Clinical genetic laboratories

#### Cytogenetic laboratories

The cytogenetics laboratories provide two types of services: clinical diagnosis and prenatal diagnosis. All but one of the cytogenetics laboratories are integral parts of the Medical genetic departments and are found in hospitals. The exception is a large laboratory of the Maccabi Health fund that provides public tests as well an important portion of the private prenatal tests in Israel. The directors of the cytogenetics laboratories are either acreditated Ph.D. medical cytogeneticists or Israeli board certified physician medical geneticists. These laboratories are regulated by the Ministry of Health.

Chromosomal microarray analysis (CMA) is performed using different platforms in several of the cytogenetics laboratories. All these laboratories offer CMA for clinical diagnosis; a few of them also perform CMA for prenatal diagnosis.

Cytogenetics for oncology/hematology are done either in general cytogenetics laboratories or in separate specialized laboratories found within the oncology/hematology departments. The set up is different from one hospital to the other.

#### Molecular genetic laboratories

While almost all the genetic departments/units have a molecular laboratory, many laboratories are independent and found outside of the genetic departments. As a rule the laboratories that are found in other hospital departments perform tests for diseases specific to that department (such as hematology or endocrinology). There are two private molecular genetic laboratories providing molecular testing including genetic sequencing.

The directors of the molecular laboratories are either acreditated Ph.D. medical molecular geneticists or Israeli board certified physician medical geneticists or Israeli board certified physicians specialized in the field of the laboratory. These laboratories are regulated by the Ministry of Health and the list of tests provided must be listed in the Israeli genetic database.

As of January 2014 next-generation sequencing is not yet performed as a clinical service in any of the Israeli accredited laboratories. Next-generation sequencing tests that are performed for medical diagnosis are done in laboratories outside of Israel including whole exome sequencing.

## Genetics Professionals in Israel

### Physicians

Since 1986 genetics is a medical specialty recognized by the Israeli Medical Association and the Ministry of Health. A two and a half year training program for medical genetics in an approved medical genetic department is available for specialists (physicians after at least 5 years training and board examination). The syllabus includes 24 months in the genetic clinics and 6 months in a genetic laboratory. After completion of training there is a national board examination including a written and an oral test.

### Genetic counselors

To become a genetic counselor in Israel one must have completed a master in human genetics or in genetic counseling. In both cases after obtaining the master degree the candidate needs to perform a 2-year training syllabus in a medical genetic department and pass a board examination.

There are two Master's degree genetic counseling training programs in Israel. The Hebrew University – Hadassah Medical School, Jerusalem program was established in 1997 and the other program that was established in 2009 in the Rappaport medical school, Technion Haifa. Both are 2-year programs with one class of six students at a time. Most of the graduates have remained in Israel to provide genetic counseling services (Sagi and Uhlmann 2013).

### Other medical geneticists

The Ministry of Health delineated professional criteria for Ph.D. medical cytogeneticists, molecular and biochemical geneticists and recognized them as specialties in health care. A separate 2-year training syllabus and a board examination exist for each these specialties since 2006. A Ph.D. medical geneticist may become director of a laboratory in his field of speciality.

## The National Program for the Detection and the Prevention of Birth Defects

The Ministry of Health sponsors a national program for the detection and the prevention of birth defects since 1980. The program is completely free of leftge and includes newborn screening, population carrier screening, and prenatal diagnosis.

### Neonatal screening

In Israel it is mandatory to offer screening to all the newborns while the parents have the possibility to opt out. The national laboratory for newborn screening is part of the community genetic department in the Ministry of Health and is located in the Sheba Medical Left, Tel Hashomer. All the data including week of pregnancy and birth weight of every child born in all the hospitals in Israel are transferred daily electronically to the central laboratory. The blood samples that are taken at least at the age of 36 h are collected by a rapid courier service allowing a very short transit time to the laboratory. When the samples arrive to the laboratory all the details about the newborn are already in the system allowing tailoring the tests according to the week of pregnancy and/or weight. Up the end of 2008, the neonatal screening included only PKU and congenital hypothyroidism. Since then the screening was expanded according to the recommendations of a national committee and includes in addition to PKU and congenital hypothyroidism, adrenal hyperplasia, and eight metabolic diseases (maple syrup urine disease, homocystinuria, tyrosinemia type I, glutaric aciduria type I, methyl malonic academia, propionic academia, medium chain acyl coA dehydrogenase deficiency, very long chain acyl coA dehydrogenase deficiency).

### National population carrier screening for reproductive purposes

Following the screening program to prevent Tay-Sachs disease among Ashkenazi Jews initiated in 1971 in the United States by Kaback (2001), a similar program was organized and funded by the Ministry of Health in Israel. In parallel, a national carrier screening program for the prevention of *β*-thalassemia has been implemented for the Arab population and some of the Jewish communities in which the disease is relatively frequent (Koren et al. 2002).

With the possibility to perform a molecular diagnosis of many genetic diseases frequent in the different parts of the Israeli population, many disorders became candidates for screening. The Association of Israeli Medical Geneticists made recommendations to include in the carrier screening program for reproductive purposes all the severe genetic diseases in which the carrier frequency is 1:60 and/or if the disease frequency higher than 1:15,000 live births. However, because of financial restrains their inclusion in the national program sponsored by the Ministry of Heath has been delayed up to 2013. Meanwhile, the screening tests have been done privately and mostly partially paid by complementary insurances.

The expansion of the national population genetic screening program has been progressive. The first step of the expansion was the addition of a program aimed to diseases found in a frequency of 1:1000 live births or higher that was initiated in 2002 (Zlotogora et al. 2009). Such high frequency of autosomal recessive severe genetic diseases is found in Israel only among Arabs and Druze. In most cases the high incidence is limited to a small community: a single locality or a Bedouin tribe. This targeted screening program included at its peak 52 different diseases in 23 different localities and all the Bedouin tribes in the Negev. Toward the end of 2008, the national program was further expanded to include cystic fibrosis carrier screening to the whole population and familial dysautonomia among Ashkenazi Jews. The final expansion of the program was in 2013. Since January 2013, the national program includes all the severe diseases in which the carrier frequency is 1:60 and/or the disease frequency is higher than 1:15,000 live births (Table 2). The program is targeted to populations at risk and includes free of leftge, carrier screening for cystic fibrosis, fragile X, and spinal muscular atrophy for most of the population and all the tests for severe diseases with a carrier frequency of 1:60 or higher. These additional tests are performed among Jews according to the community of origin and among Arabs and Druzes according to the religion and the locality of origin or according to the tribe among the Bedouins of the Negev.

**Table 2:** The national population carrier screening for reproductive purposes.

1. *Genetic screening among Jews*
Cystic fibrosis
Fragile X
SMA
Screening limited to Jews from a specific origin
Ashkenazi Jews
Tay Sachs, familial dysautonomia
Canavan disease
North African Jews
Thalassemia, Tay Sachs
Jews from Morocco
Progressive cerebello cerebro atrophy
Jews from Yemen
Metachromatic leukodystrophy (MLD)
Jews from Iraq
Thalassemia, MGA3.
Progressive cerebello cerebro atrophy
Jews from the Mediterannean region, Kurdistan, Iran or Asiatic countries from the former USSR
Thalassemia
Jews for the Balkan
Familial dysautonomia
Jews for the Caucasus
Infantile cerebral cerebellar atrophy (ICCA)
2. *Genetic screening among Christian Arabs*
Cystic fibrosis
Fragile X
SMA
Thalassemia
Cockayne syndrome
Albinism
Screening limited to Christians Arabs in a specific locality
Ataxia telangiectasia
Sandhoff
3. *Genetic screening among Muslim Arabs (not including the Bedouins)*
Cystic fibrosis
Fragile X
SMA
Thalassemia
Screening limited to the Muslim Arabs in a specific locality
Ataxia telangiectasia
Bartter and Gitelman syndrome
Biotinidase deficiency
Cerebral dysgenesis, neuropathy, ichthyosis and keratodermia (CEDNIK)
Cockayne/XP
Complex hereditary spastic paraparesis
Congenital insensitivity to pain with anhidrosis
Congenital nephrotic syndrome
Congenital thyroid hormone and glucocorticoid deficiency
Epidermolysis bullosa
Factor 7 deficiency
Glutaric aciduria type II
Gray platelet syndrome
HGM
Hyperuricemia, pulmonary hypertension, renal failure, and alkalosis (HUPRA)
Hyperoxaluria
Hypoparathyroidism, retardation, dysmorphism
Hypophosphatasia
Infantile bilateral striatal necrosis (IBSN)
Krabbe disease
Leber amaurosis
Limb girdle muscular dystrophy
Mental retardation, non syndromic
Mitochondrial DNA depletion syndrome
Molybdenum cofactor deficiency
Non ketotic hyperglycinemia
Pelizaeus-Merzbacher like disease
POC1A deficiency
Pompe disease
Pseudo rheumatoid dysplasia
Pycnodysostosis
Retinitis pigmentosa
Rickets, 1,25 dehydroxy vitamin D3 resistant
Severe combined immune deficiency
Smith Lemli Opitz
Spinal muscular dystrophy-related disease
Stuve Wiedemann
Ventricular tachycardia
Wilson disease
Wolman disease
4. *Genetic screening among the Negev Bedouins*
SMA
Hypoparathyroidism, retardation, dysmorphism
Congenital insensitivity to pain
Screening according to the tribe
Arthrogryposis
Ataxia telangiectasia Bardet Biedl syndrome
Bartter syndrome
Cardiomyopathy dilated, neonatal isolated
Carmi syndrome-Epidermolysis bullosa, pyloric stenosis
Carnitine-acylcarnitine translocase deficiency
Complex III deficiency, mitochondrial respiratory chain
Cornelia de Lange like (Birk Flusser) syndrome
Cystinuria + (2p16 del) syndrome
Cystic fibrosis
Desmosterolosis
Glycogen storage disease
Growth hormone deficiency
Hemolytic uremic syndrome (Complement H factor 1 deficiency)
Infantile bilateral striatal necrosis (IBSN)
Infantile neuroaxonal dystrophy (INAD)
Infantile sialic acid storage disease (ISSD)
Leber's congenital amaurosis
Maple syrup urine disease (MSUD)
Nephronophthisis
Niemann Pick type C
Non ketotic hyperglycinemia
Osteogenesis imperfecta
Osteopetrosis
Pelizaeus-Merzbacher-like syndrome
Persistent hyperinsulinemic hypoglycemia of infancy (PHHI)
Pyruvate dehydrogenase deficiency (PLD)
Thalassemia
5. *Genetic screening among the Druze*
Cystic fibrosis
Fragile X
SMA
Thalassemia
Screening limited to the Druze in a specific locality
Argininosuccinic aciduria
Ataxia telangiectasia
Carbamoyl phosphate synthetase I deficiency
Cerebrotendinous xanthomatosis
Cockayne syndrome
CPTII
Fanconi A
Hyperoxaluria
Kohlschütter-Tönz disease
Krabbe disease
Maple syrup urine disease
Mitochondrial DNA depletion syndrome
Mucolipidosis III
Pompe disease
Prolidase deficiency
Retinitis pigmentosa
Tay Sachs disease
Thyrosinemia
Wilson disease

Most of the tests are performed before/during pregnancy. The tests are sequential, most often the woman is first tested and if found to be a carrier her partner is also tested. The tests are performed either in the medical genetic departments/units or in the community under the responsibility of a physician medical geneticist. The recommendations and explanations about the tests are given either by genetic counselors or by individuals from the medical personnel that received appropriate tutoring under the responsibility of the medical geneticist in leftge. If a carrier is diagnosed the results are explained by a genetic counselor. Couples at risk receive genetic counseling and may chose any of the reproductive options free of leftge. Couples from the ultra orthodox community may have the tests through the Dor Yeshorim program.

In 2013, the first year of the expanded program, ∼60,000 individuals were examined and 270,000 tests were done.

### The Dor Yeshorim program

In the ultraorthodox Jewish Ashkenazi community, prenatal diagnosis is not an acceptable mean of prevention because of religious beliefs against abortion. Therefore, a special program for carrier screening prior to marriage has been implemented (Ekstein and Katzenstein 2001). The purpose of this approach is to prevent mating of two heterozygotes of the same gene. Since in this community the marriages are “arranged” by the families this genetic testing has become the precursor of the engagement. In addition, the screening is done anonymously and the results given are only whether the marriage is possible or not. The preservation of anonymousity was a very important precondition for this community and is one of the main reasons for the success of the program.

### Prenatal diagnosis

In 1980, the Ministry of Health began to offer amniocentesis free of leftge, to all pregnant women over the age of 37 years at the time of their last menstrual period and since 1993 the age was reduced to 35. In 2014, a woman is eligible to free amniocentesis either if she is older than 35 or if the test is recommended by a medical geneticist because of an increased risk either for a chromosomal aberration or for a monogenic disease. From May 2012, women with the diagnosis of a fetal malformation by ultrasound are entitled to free CMA fetal examination.

## Financement of Genetic Services in Israel

### Diagnosis and genetic counseling

Genetic tests are available free of leftge if recommended by a medical geneticist when the indication is well-defined and if the test is done in Israel. The criteria for performing genetic tests free of leftge are very specific and therefore limited.

### Prenatal diagnosis

Prenatal diagnosis is free of leftge for women at 35 year of age at the time of their last menstrual period or if the test is recommended under specific conditions, by a medical geneticist. Other women may have the prenatal diagnosis done privately (or paid by for by available complementary insurances).

PGD is available free of leftge for couples at risk for a severe genetic disease. Non invasive prenatal test (NIPT) is available privately, partially covered by complementary insurance being performed in the different laboratories outside of Israel.

### Genetic screening

The tests recommended by the Association of Israeli Medical Geneticists are available free of leftge. Other screening tests may be done as part of the complementary insurance or privately.

## Some Reflexions

In the debate regarding disability rights and prenatal diagnosis, the majority of the Israeli population is in favor of prenatal genetic testing as well as selective abortion (Raz 2004). In Israel, people with disabilities in particular those born with genetic diseases often face difficulties in many aspects of life even though the Israel's Equal Rights for People with Disabilities Law promotes their right to equality. Prevention of severe genetic diseases and malformations using carrier screening and/or prenatal diagnosis is deeply anchored in the medical community dealing with pregnancy. Genetic carrier screening for reproductive choices that was first introduced in Israel for Tay Sachs disease is responsible for its almost disappearance among Jews. Later, probably as a direct result of the overwhelming success of the Tay Sachs program, Israel was the first country in which population screening was introduced for cystic fibrosis, fragile X, and spinal muscular atrophy. The national program is to my knowledge unique offering not only screening for cystic fibrosis, fragile X, and spinal muscular atrophy free of leftge to all the population but also many disorders that are tested according to the ethnicity and religion. While in many countries such emphasis on the ethnicity and religion may be subject to debate or to rejection, in Israel it has been well-accepted. This may be related to the fact that in Israel, the distinction between Jews and Arabs and within the Jewish population between Ashkenazi and others Jews according to their country of origin is an integral part of the daily life. Details about the ethnicity and religion are routinely used in medical genetics either for Jews, Arabs, or Druzes without any debate or questions about discrimination either within the medical community or the general public.

Another field that has been in continuous expansion is the use of prenatal screening and testing with the aim of prevention. The use of prenatal screening tests including those recommended or those done privately is very high in particular in the nonorthodox Jewish population and the non-Jewish population (Sher et al. 2003, 2004). Lately, as expected the demand private of fetal chromosomal screening using NIPTs has been has been high (no data available). Relatively high-utilization rates of invasive prenatal diagnostic tests is seen mainly by the nonorthodox Jewish population who as already discussed will often use the possibility to terminate the pregnancy because of a fetal disease or malformation (Sher et al. 2003). While termination of pregnancies are allowed in Israel only according to definite criteria, the one concerning malformations and or genetic diseases is vague and may be accepted by the medical committee even in cases of relatively mild medical problems in particular before the 22 weeks of pregnancy. Later abortions are possible but only when there is a significant risk of a severe disease in the fetus.

In contrast to the prevention, there have been almost no public interest and or/debate about neonatal screening. The expansion of the newborn screening program has been promoted by the Ministry of Health without any pressure either of the medical community or the general public. Only recently, the physicians in leftge of cystic fibrosis and severe immune deficiency (SCID) patients have been active trying to introduce these diseases in the neonatal screening in the newborn screening program. Screening for cystic fibrosis was not included in the expansion of the program in 2009 because of the relatively small number of children born affected with the disease as a direct consequence of the population carrier screening (Zlotogora and Israeli 2009). The screening for SCID has been recommended since 2012 by the committee following the program but has not been yet included because of financial constrains.

## Biosketches

Joël Zlotogora, M.D., Ph.D., is an adjunct professor of human genetics at the Hebrew University Jerusalem. Jëel Zlotogora is a pediatrician who has completed a fellowship in medical genetics and a Ph.D. in biochemical genetics in the genetic department in Haddasah Medical Center where he was a senior physician till 1999 when he moved to the Ministry of Health. Since 2000, Dr. Zlotogora is the Director of the Department of Community Genetics in the Ministry of Health and is responsible for the genetic services in Israel and in particular for the national program for the detection and the prevention of birth defects.
